# Choice of Allograft in Patients Requiring Intestinal Transplantation: A Critical Review

**DOI:** 10.1155/2017/1069726

**Published:** 2017-05-03

**Authors:** Genevieve Huard, Thomas Schiano, Jang Moon, Kishore Iyer

**Affiliations:** ^1^Intestinal Rehabilitation and Transplantation Program, Recanati/Miller Transplantation Institute, Mount Sinai Medical Center, New York, NY, USA; ^2^Division of Liver Diseases, Mount Sinai Medical Center, New York, NY, USA; ^3^Division of Liver Diseases, Centre Hospitalier de l'Université de Montréal, Montréal, QC, Canada

## Abstract

Intestinal transplantation (ITx) is indicated in patients with irreversible intestinal failure (IF) and life-threatening complications related to total parenteral nutrition (TPN). ITx can be classified into three main types. Isolated intestinal transplantation (IITx), that is, transplantation of the jejunoileum, is indicated in patients with preserved liver function. Combined liver-intestine transplantation (L-ITx), that is, transplantation of the liver and the jejunoileum, is indicated in patients with liver failure related to TPN. Thus, patients with cirrhosis or advanced fibrosis should receive a combined allograft, while patients with lower grades of liver fibrosis can usually safely undergo ITx. Reflecting their degree of sickness, the waitlist mortality rate and the early posttransplant outcomes of patients receiving L-ITx are worse than IITx. However, L-ITx is associated with better long-term graft and patient survival. Multivisceral transplantation (MVTx), that is, transplantation of the organs dependent on the celiac axis and superior mesenteric artery, can be classified into full MVTx if it includes the liver and modified MVTx if it does not. The most common indications for MVTx are extensive portomesenteric thrombosis and diffuse gastrointestinal pathology such as motility disorders and polyposis syndrome. Every patient with IF should undergo a multidisciplinary evaluation by an experienced ITx team.

## 1. Introduction

Intestinal failure (IF) is defined as a critical reduction of the functional gut mass leading to the inability to maintain fluid, electrolyte, and protein-energy balance such that intravenous supplementation becomes necessary [1–3]. The development of successful intestinal and multivisceral transplantation is one of the most recent milestones in the field of intestinal rehabilitation and solid organ transplantation [4]. Currently, intestinal transplantation (ITx) is offered to patients with irreversible IF and total parenteral nutrition (TPN) failure in which survival on TPN is compromised [2, 5, 6]. TPN-related complications recognized as indications for ITx by the Center for Medicare and Medicaid Services are the following: impending or overt liver failure related to TPN (parenteral nutrition associated liver disease (PNALD)), impending loss of vascular access for TPN administration, multiple episodes of catheter-related sepsis, a single episode of life-threatening catheter-related sepsis, or frequent episodes of significant dehydration despite supplemental fluid administration (Table 1) [2, 7–9].

In the past decade, the outcomes of ITx have greatly improved. As shown in the most recent report from the Intestinal Transplant Registry (ITR) (worldwide data), the current 1-, 5-, and 10-year patient survival rates after ITx are 77%, 58%, and 47%, respectively [10, 11]. Moreover, the current 1-, 5-, and 10-year graft survival rates are 71%, 50%, and 41%, respectively [10, 11].

**Figure 1:**
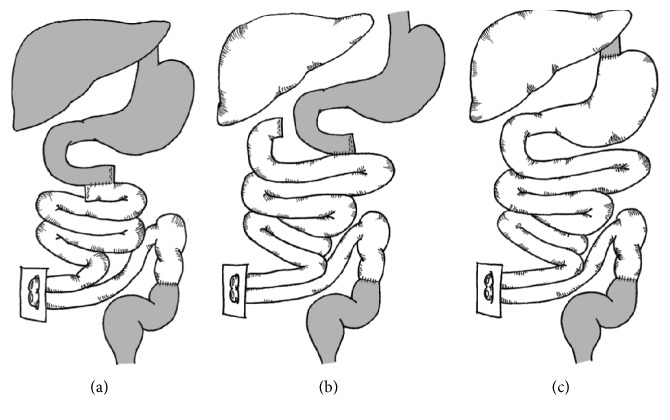
Types of allograft in intestinal transplantation. (a) Isolated intestine transplantation (IITx), (b) combined liver-intestine transplantation (L-ITx), and (c) multivisceral transplantation (MVTx).* Note*. Organs in grey represent native organs and dashed organs represent transplanted organs.

The jejunoileum is the defining component of any ITx. Depending on the inclusion of other organs along with the jejunoileum, ITx can be classified into three main categories: isolated intestinal transplantation (IITx) with or without colon, combined liver-intestine transplantation (L-ITx), and multivisceral transplantation (MVTx) [12] (Figure 1). While controversy persists regarding nomenclature, MVTx may be defined as the replacement of organs depending on the celiac trunk and superior mesenteric artery (SMA), that is, the stomach, liver, pancreas, duodenum, jejunum, and ileum [12]. MVTx can be “full” if it includes the liver or “modified” (MMVTx) if it does not include the liver [12]. According to the most recent report from the ITR, from 2001 to 2011, 46.6% of ITx were IITx, 26.6% were combined L-ITx, 21.3% were MVTx, and 5.5% were MMVTx [10, 11]. More recently, the proportion of ITx including the liver as part of the allograft is greatly decreased. Forty-eight percent of the allografts included the liver between 2001 and 2011 as compared to 61.8% and 53.2% between 1985–1995 and 1995–2001, respectively [10, 11].

The choice of intestinal allograft depends on many factors including the underlying pathology leading to irreversible IF, the absence or presence of simultaneous organ dysfunction (e.g., chronic renal failure, diabetes, and liver failure), age and size of the patient (there are donor-recipient match size issues in smaller patients), anatomy of the recipient, organ availability in the different organ procurement organizations, and transplant center expertise or preferences [12]. Currently, there is no published consensus regarding the choice of allograft in patients requiring ITx. Herein, we critically review the current literature regarding the complex decision-making process deciding on the type of allograft needed in patients requiring ITx.

## 2. Intestinal Failure and Intestinal Rehabilitation

Soon after an extensive intestinal resection, several physiological adaptation mechanisms such as increased villous height and crypt depth as well as intestinal dilatation are initiated and continue over the first two years in order to restore nutritional autonomy [1, 13, 14]. Usually, these adaptation mechanisms are sufficient to enhance the absorptive surface area of the residual small bowel (SB) and intravenous nutritional support is therefore temporary in the majority of the cases [1]. In cases in which these adaptive mechanisms are insufficient, nutritional dependency becomes irreversible and long-term TPN is the standard therapy [15, 16]. In such circumstances, intestinal rehabilitation should be attempted in order to safely reduce the need for TPN and therefore avoid the complications related to long-term TPN. Intestinal transplantation should only be considered after failure of dedicated attempts at intestinal rehabilitation and after the development of life-threatening complications related to TPN [2, 5, 7].

Given the complexity of the management of patients with IF, such patients should be referred to and followed by a multidisciplinary program with expertise in IF and intestinal rehabilitation [17]. Early referral to such a program is associated with better outcomes, reduced morbidity, and reduced mortality in patients with IF [7, 18].

The first step of intestinal rehabilitation is usually proper dietary management and adjunctive medications [20, 21]. In general, such patients benefit from hyperphagia to compensate for their malabsorptive state. Moreover, they should be encouraged to have small and frequent meals to improve absorption [15]. Patients with colon in continuity with SB usually benefit from a diet rich in complex carbohydrates and low in fat [15, 22]. Finally, these patients should be encouraged to limit their fluid intakes and to consume isoosmotic fluids such as the oral rehydration solution in order to optimize water and sodium absorption [15, 21, 23].

In addition to dietary modifications, several medications are recommended in patients with IF in order to avoid dehydration associated with high stoma outputs. Proton pump inhibitors are usually recommended in the first few months following an extensive intestinal resection in order to compensate for the hypergastrinemia state and the consequent gastric acid hypersecretion [24, 25]. The use of antidiarrhoeal medications such as loperamide is standard in patients with IF in order to increase intestinal transit time and therefore improve absorption and reduce stoma output [15]. When clinically appropriate, enterocyte growth factors should be considered to promote the physiological adaptation mechanisms [14]. Recently, recombinant human glucagon-like peptide- (GLP-) 2 analog (teduglutide) has been shown to reduce the volume and the number of days of TPN support [26–28]. The underlying mechanism of action of teduglutide probably involves mucosal growth by promoting intestinal crypt cell proliferation and inhibiting apoptosis [29]. Moreover, GLP-2 analogs have been shown to increase intestinal transit time and decrease gastric emptying and gastric acid secretion [30].

Finally, several surgical techniques are now available for patients with irreversible IF. Whenever possible, the continuity between the SB and the colon should be restored in order to improve fluid and energy balance [15]. When clinically appropriate, surgical lengthening procedures (i.e., STEP procedure nad Bianchi procedure) should be considered before referral for intestinal transplantation [31, 32].

Given all these recent improvements in the management of patients with IF, intestinal transplantation should therefore only be considered in the minority of patients who failed dedicated attempts at intestinal rehabilitation.

## 3. Isolated Intestine Transplantation

IITx is indicated for patients with irreversible IF and preserved liver function [12, 33]. Currently, the most common indications for IITx in patients with irreversible IF are impending vascular access loss for TPN administration and repeated or life-threatening line sepsis [2]. IITx should only be considered in patients who failed dedicated attempts at intestinal rehabilitation.

The venous drainage of the allograft may be achieved through orthotopic portal drainage but is more usually achieved systemically through direct anastomosis to the inferior vena cava (IVC). The initial attempts to drain isolated intestinal allografts systemically came in patients receiving IITx in the presence of moderate degrees of liver fibrosis, where systemic venous drainage was considered safer by avoiding venous anastomosis against higher vascular resistance. In the published Miami and Pittsburgh experiences, systemic compared to portal drainage was not associated with increased risk of patient or graft loss [34, 35]. However, in the Miami series, systemic drainage was associated with an increased risk of Gram-negative bacteremia and pneumonia, possibly reflecting the important role of the liver in the clearance of translocated intestinal bacteria [34]. Interestingly, systemic drainage was not associated with increased risk of hepatic encephalopathy [34], although at our center we have had 3 (unreported) cases of episodic hyperammonemia and concomitant mental status changes in patients whose isolated intestinal allografts were drained systemically. The Pittsburgh experience also revealed that systemic drainage was not associated with an increased risk of rejection [35]. Thus, the technically easier systemic venous drainage does not appear to be associated with significant adverse outcomes [34, 35].

In patients suffering concomitantly from pancreatic insufficiency, such as in patients with type 1 diabetes or cystic fibrosis, the pancreas can also be included in the allograft, either as a composite graft or simultaneous implantation of the intestine and pancreas from the same donor [12]. The indications for combined intestinal and kidney transplant are not as well defined in the current literature. Clearly, patients on long-term hemodialysis at the time of evaluation for IITx should be evaluated for concurrent kidney transplant [36]. Also, given that the incidence of chronic renal failure is greater than 20% five years after ITx, patients with abnormal renal function going into transplant should be evaluated for possible concurrent kidney transplantation [37, 38]. Avoidance of early renal insufficiency is of major importance given that it will have a significant impact on immunosuppression and fluid management in the posttransplant setting. Moreover, as shown in the published experience from Los Angeles, renal dysfunction (GFR < 75% of normal) at day 7, 1 month, and 1 year after ITx was associated with an increased risk of death (HR: 1.5, 1.2, and 6.0, resp.) [39].

In some centers, the right hemicolon (vascularized by the SMA) and the ileocecal valve are now routinely included in the allograft of patients undergoing IITx [12, 40]. In an early report of 71 ITx, including 29 colon-containing allografts, inclusion of the colon appeared to be significantly associated with decreased graft and patient survival [41]. However, these early findings were not subsequently confirmed and the practice of colon inclusion has resumed in some centers since the publication of the Paris and the Miami experiences [40, 42]. In the Paris series, 23/36 children received an allograft including the colon (17 were L-ITx) and colonic inclusion had no impact on patient or graft survival [42]. Kato et al. reported on 93 ITx that included the colon out of a total of 245 ITx [40]. Inclusion of the colon was not associated with decreased graft or patient survival [40]. Moreover, colonic inclusion was associated with a higher frequency of formed stools after stoma closure (67% in patients with colon inclusion versus 48.5% in patients who did not receive a colon as part of the allograft) [40]. Since the publication of those two recent series, it is now believed that colonic inclusion has no unfavorable impact on the posttransplant outcomes of ITx. According to the most recent ITR report, in 2012, 30% of the intestinal allografts included the colon in comparison to 4% in 2000 [10]. Colonic inclusion may improve quality of life after transplantation, being associated with less diarrhea after stoma reversal. Moreover, as demonstrated in the ITR report, colonic inclusion increased the likelihood of being free from TPN and intravenous fluid supplementation by 5% in comparison to those who did not receive a colonic segment, reflecting the important role of the colon in fluid and free fatty acid absorption [10]. In this same report, colonic inclusion did not increase the risk of rejection after transplantation [10]. Of note, the inclusion of colon in the allograft does not allow the performance of ITx without a temporary ileostomy. The presence of a temporary ileostomy is essential for easy access to the allograft for endoscopic monitoring after transplantation. The inclusion of the ileocecal valve may increase the difficulty in endoscopically accessing the ileal component of the graft after ITx and, thus, a temporary ileostomy remains essential [40]. In the Miami series, there were no cases of acute cellular rejection (ACR) restricted to the colonic segment of the allograft [40].

The intestinal allograft is highly immunogenic and chimeric, containing a large amount of lymphoid tissue (gut-associated lymphoid tissue) with the genotype of the epithelial cells remaining mainly that of the donor [2, 35, 43]. The outcomes of IITx are thus marked by high rates of ACR [35]. Also, the rates of ACR after IITx are higher than those seen after L-ITx or MVTx, that is, after transplantation of a liver-containing allograft [35, 44–47]. It is thought that the transplantation of the liver in association with the intestine promotes tolerance towards the bowel graft by inducing the production of regulatory T-cells and the deletion of alloreactive T-cells [19, 35, 48]. A multivisceral allograft might confer even more protection against severe ACR of the intestinal allograft in comparison to L-ITx [46]. MMVTx, that is, allograft not including the liver, may also confer protection against ACR in comparison to ITx [44]. One theory to explain this finding is that the risk of ACR might be related to the relative proportion of donor lymphoid tissue transplanted with the allograft compared to the remaining recipient lymphoid tissue [44]. MVTx and MMVTx come by default with a larger amount of lymphoid tissue and may thus be more capable of inducing tolerance towards the intestinal allograft [44]. In the preinduction era, ACR of the intestinal allograft in the first 30 days after transplantation was reported to be as high as 88% [49]. Currently, according to the most recent report from the Scientific Registry of Transplant Recipients (SRTR), the cumulative incidence of ACR after ITx is 39% at 12 months and 44% at 24 months [19].

## 4. Combined Liver-Intestine Transplantation

L-ITx is indicated for patients with irreversible IF despite intestinal rehabilitation and impending or overt liver failure related to TPN (PNALD) [2, 12, 33, 50–52]. Usually, the liver is transplanted en bloc along with the pancreas and small bowel in order to avoid hilar dissection and decrease the risk of vascular and biliary complications after transplantation [6, 53, 54].

Historically, ITx was most commonly performed in combination with a liver allograft, since most patients listed for ITx also had advanced PNALD. As per the 2005 United Network for Organ Sharing (UNOS) data, 74% of the patients listed for ITx also required listing for a liver transplant, either before (10%), simultaneously (52%), or after (12%) an ITx [52]. Since 2008, proportionally less L-ITx are performed compared to IITx [19]. According to the last SRTR report, in 2012, only 42% of ITx included a liver allograft [19]. This recent reduction in the number of L-ITX performed annually is most likely a reflection of the advances in the understanding of the physiopathological mechanisms of PNALD and of the improved care of patients on long-term TPN. In addition to TPN composition optimization and lipid restriction, fish oil emulsions (Omegaven) are now available in many countries [55, 56]. Replacement of the soy-based lipid emulsions by omega-3 rich lipid emulsions is associated with rapid improvement of cholestasis in the majority of the cases [56–58]. However, whether this biochemical cholestasis reversal is associated with hepatic fibrosis regression remains highly controversial [55, 56, 58–61].

Currently, there is no clear consensus defining when a liver should be included in the allograft [50]. Patients on TPN who develop cirrhosis have essentially a 100% mortality rate at 5 years without transplantation [52, 62]. Chan and coworkers reported 6 individuals with end-stage liver disease (ESLD) related to TPN with universal mortality, occurring at a median of 10.8 months following the first elevation of bilirubin [63]. Another study showed a 1-year and 2-year survival rates of only 30% and 22%, respectively, in patients listed for L-ITx who did not undergo ITx [64]. Given these results, it is generally accepted that individuals with cirrhosis secondary to TPN and those with advanced stages of fibrosis should be considered for concurrent liver transplantation [12, 33, 50–52]. As a general rule, patients with biopsy-proven advanced fibrosis (F3-F4) and those with clinical signs of cirrhosis (portal hypertension, coagulopathy) should not receive an IITx. In the cases of lesser grades of fibrosis, PNALD may regress or resolve after a successful IITx allowing for weaning and eventual withdrawal of TPN [12, 50, 51]. In one series, four patients having liver fibrosis stage 2 or 3 underwent IITx. Nine months after transplant, all patients showed regression of liver fibrosis with an improvement of at least one stage on posttransplant liver biopsy [65]. Moreover, early cirrhosis reversal 17 months after a successful IITx and TPN weaning has been demonstrated [66]. Since evaluation of the extent of PNALD may be challenging, a liver biopsy should be performed prior to ITx in patients with persistent elevation of the total bilirubin and/or low platelet counts in order to guide the choice of allograft. When possible, a transjugular liver biopsy should be chosen over a percutaneous liver biopsy, since this procedure also allows for measurement of the portal pressure gradient, recognizing that, in the patient with short bowel, wedge pressure measurements may grossly underestimate actual portal venous pressures. Massive resection of the small bowel and thus decreased portal venous return to the liver can mask the presence of advanced fibrosis with clinical portal hypertension not occurring. Hence, patients with extreme short bowel syndrome may not manifest the usual stigmata of ESLD in the form of ascites or esophageal varices, and synthetic dysfunction is a very late and poor prognostic sign. In these patients, presence of hepatosplenomegaly, thrombocytopenia, and engorged superficial abdominal veins may be the only signs of ESLD with portal hypertension and constitute absolute indications for liver replacement in addition to the intestine.

**Figure 2:**
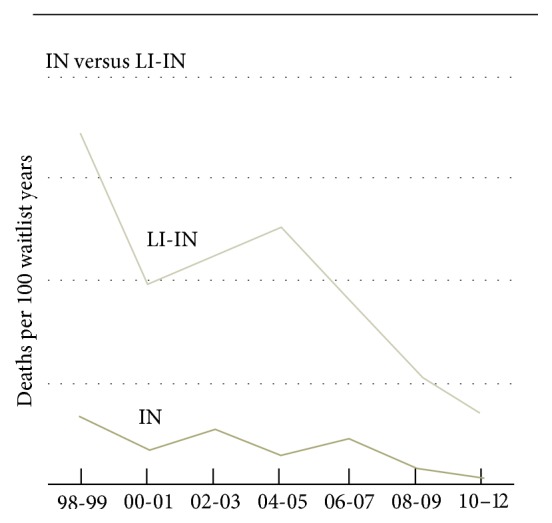
Waitlist mortality among patients listed for isolated intestinal transplantation and combined liver-intestine transplantation.* Notes*. (1) From SRTR report 2012 [19]. (2) LI-IN: combined liver-intestine transplantation; IN: isolated intestinal transplantation.

An important consideration in the choice of allograft is the higher mortality on the waiting list in patients waiting for L-ITx compared to those listed for IITx [19, 50, 52, 62, 67–69]. Since the creation of the waiting list for ITx in 1994, the mortality in candidates listed for L-ITx has greatly exceeded that of all other solid organ transplant candidates [50, 67]. The worse short-term prognosis of patients listed for L-ITx was not appropriately taken into account in the previous organ allocation systems and these patients were thus disadvantaged compared to other liver transplant (LT) candidates [62, 68, 69]. Prior to 2002, it was estimated that 86% of all deaths occurring in patients listed for ITx were in those waiting for L-ITx [62]. Also, children seemed to be disproportionately affected given that 83% of all deaths were in pediatric candidates [62]. In February 2002, the MELD and PELD scores were implemented to better prioritize patients at higher risk of short-term mortality on the waiting list [69, 70]. Despite the fact that MELD and PELD scores are based on objective criteria, candidates listed for L-ITx were unfortunately still at higher risk of death and less likely to be transplanted compared to their counterparts listed for LT or IITx [71, 72]. To account for the higher mortality risk in patients with IF and PNALD listed for L-ITx, modifications to the MELD and PELD scoring systems were implemented in 2005 and 2007 [69]. Currently, when an adult's MELD score does not adequately reflect the severity of his condition, an appeal for extra MELD points can be filed with the Regional Review Board (UNOS policy 3.6.4.1 Adult Candidate Status). For children below 12 years and adolescents aged between 12 and 17 years listed for L-ITx, extra 23 points are currently added to their natural PELD score to account for their higher mortality risk on the waiting list [69]. Moreover, in 2005, a status IB was created for pediatric candidates waiting for L-ITx [69]. Following these modifications to the MELD and PELD scores, Kaplan et al. assessed the impact of these modifications on the waiting list mortality of L-ITx candidates [69]. According to their analysis of UNOS data from 1999 to 2009, the policy modifications were highly effective in eliminating the mortality disparities on the waiting list for adults with a MELD score over 15 points [69]. However, adult patients listed for L-ITx with MELD score under 15 points were still disproportionately at higher risk of mortality compared to their LT counterparts [69]. This same study revealed an improvement in the waiting list mortality for pediatric candidates but the policy revisions were not sufficient to completely eliminate the mortality disparities between L-ITx and isolated LT pediatric candidates [69]. According to the most recent SRTR report, 11.6% (6.7 deaths/100 waitlist years in 2010–2012 versus 51.0/100 waitlist year in 1998-1999) of ITx candidates died on the waiting list in 2012 [19]. Despite the recent improvement in the waitlist mortality for L-ITx, in 2012, the waiting list mortality rate was still almost 10 times higher for L-ITx candidates (14.2 deaths/100 waitlist years) versus IITx candidates (1.5 deaths/100 waitlist years) (Figure 2) [19]. Regional disparities in PELD/MELD scores at the time of transplantation and waitlist mortality continue to be significant, forcing a need to search for alternative strategies to tackle this issue [50, 52, 73].

IITx can sometimes be performed if the chances of survival while waiting for a L-ITx are estimated to be lower than the chances of survival after IITx and if there is an expectation that the native liver will recover after successful intestinal engraftment. Such an approach is associated with a potential risk of liver decompensation and encephalopathy after IITx. If the posttransplant course is uncomplicated and the TPN can be weaned, it appears that liver function can be preserved without liver replacement. While it is unclear whether biochemical improvement in liver function is paralleled by histological improvement, we have reported downstaging of fibrosis in a small number of patients after IITx and even reversal of TPN-related cirrhosis 17 months after IITx [65, 66].

**Figure 3:**
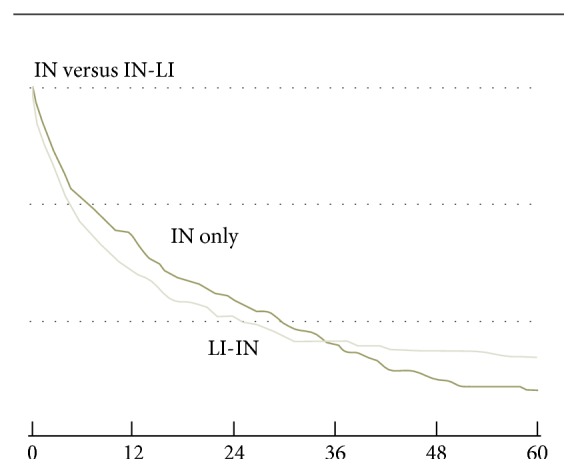
Graft survival after isolated intestinal transplantation versus combined liver-intestine transplantation.* Notes*. (1) From SRTR report 2012 [19]. (2) LI-IN: combined liver-intestine transplantation; IN: isolated intestinal transplantation.

L-ITx carries a higher early postoperative mortality rate but a better long-term graft and patient survival rates compared to IITx [52]. The higher initial mortality rate seen in L-ITx recipients is thought to be, in part, secondary to their poorer clinical status going into transplant compared to their IITx counterparts [48, 52, 62]. Moreover, when life-threatening complications such as infections or PTLD occur after transplant, the intestinal allograft can be removed and all immunosuppression ceased in the IITx recipients with TPN being reinstituted [62, 68, 74]. This life-saving option is not possible in the L-ITx recipients, making them less easily salvageable when similar complications occur after transplant [62, 68, 74]. However, as shown in the last SRTR report, 3 years after transplantation, L-ITx recipients demonstrate better long-term outcomes compared to their IITx counterparts [19] (Figure 3). These better long-term outcomes may be related to the liver-induced tolerance towards the allograft, with decreased risk of ACR and chronic rejection [35, 48].

## 5. Multivisceral and Modified Multivisceral Transplantation 

In many centers, the term MVTx is used to refer to allografts including the stomach and the duodenum in continuity with the jejunoileum [2, 12]. MVTx can also refer to the transplantation of all organs dependent on the celiac artery axis and the SMA (stomach, liver, pancreas, duodenum, and jejunoileum) [12, 50, 75–77]. This definition distinguishes MVT from L-ITx, only by the inclusion of the stomach in the former. An alternative definition that has not been widely accepted distinguishes MVTx only by the need for upper abdominal exenteration and not by the inclusion or exclusion of the stomach. Moreover, a multivisceral allograft can be “full” (MVTx) if it includes the liver and “modified” (MMVTx) if it does not [12, 50, 75–77].

The number and the type of organs included in a multiorgan allograft depend largely on the underlying pathology leading to transplant [12, 33]. Currently, the most frequent indication for MVTx is extensive portomesenteric thrombosis with hepatic decompensation and/or life-threatening bleeding complications related to portal hypertension [2, 12, 33, 50–52]. In this particular situation, the extensive vascular thrombosis surgically precludes an isolated LT [2, 12, 33, 50–52]. Furthermore, the alternate approach of isolated LT with cavoportal hemitransposition is associated with poor patient and graft survival and usually does not provide adequate decompression of the thrombosed portomesenteric and splenic systems [78]. As a result, patients can still experience complications related to portal hypertension [78]. Other common indications for MVTx are familial polyposis syndromes (familial adenomatous polyposis, Peutz-Jeghers syndrome), massive abdominal desmoid tumors, and locally aggressive benign tumors requiring total exenteration [12, 33]. Traumatic loss of abdominal viscera, such as can be seen after major motor vehicle accidents, is also an indication for MVTx [12, 33]. Diffuse gastrointestinal motility disorders, such as chronic intestinal pseudoobstruction (CIPO), scleroderma, and hollow visceral myopathy syndrome, can also warrant a MVTx [12, 33]. Of note, whenever the native liver function is preserved and it is surgically feasible, the allograft should not include the liver.

Some indications for MVTx warrant further clarifications. In the case of diffuse portomesenteric thrombosis in association with cirrhosis, the extent of the venous thrombosis usually surgically contraindicates the transplantation of an isolated liver for technical reasons and inability to provide portal venous inflow. In this situation, a full MVTx should be performed and is usually associated with excellent outcomes [79]. In the case of diffuse portomesenteric and splenic thrombosis in a noncirrhotic patient who develops life-threatening bleeding from portal hypertension, the decision to perform a MVTx should be reserved only after the patient has failed attempts at TIPS and/or surgical shunts [35, 80]. In patients having familial adenomatous polyposis, some centers advocate for MVTx given the increased risk of malignancy in the pancreaticoduodenal complex [75]. Duodenal adenomatosis, a premalignant condition, may warrant duodenectomy and pancreatectomy as an attempt to prevent malignant transformation after transplantation [75]. This surgical approach with subsequent MVTx should be considered in patients with FAP, mostly in the presence of advanced dysplastic changes, rapidly growing adenomas, and a family history of duodenal cancer [75]. For CIPO, there is no current consensus on the number and the type of organs that should be included in the allograft. Given the diffuse nature of the motility disorder, some centers advocate for the inclusion of the stomach as part of the allograft, while others perform ITx with or without hemigastrectomy and gastrojejunostomy to favor gastric emptying [81, 82].

A last point to consider in the decision to perform a MVTx is that posttransplantation outcomes are marked by increased risks of infections compared to IITx [35, 46, 83]. Moreover, it appears that the risks of CMV and PTLD are higher after MVTx, contributing to the less favorable outcomes of MVTx [83]. A recent series also reported a mildly increased risk of graft versus host disease (GVHD) compared to other types of ITx: 14% for MVTx, 10% for MMVTx, 8% for L-ITx, and 6% for ITx [35].

## 6. Isolated Liver Transplantation

Some centers have advocated isolated LT for children who develop ESLD secondary to TPN before full expected intestinal adaptation occurs. Such an approach can be justified in certain children who demonstrate a reasonable tolerance to enteral feeding, usually defined as greater than 50% of the total caloric needs, and continuous signs of progressive intestinal adaptation [84–87]. In children, the length of small bowel cannot be used to predict the likelihood of nutritional independency. However, isolated LT should not be performed when the remaining length of functioning small bowel is less than 25 cm [84, 85].

The rationale justifying the performance of an isolated LT in these children is that ESLD and portal hypertension can blunt further intestinal adaptation and can also preclude the performance of surgical lengthening procedures that could allow for TPN weaning [84–86]. Moreover, the outcomes of ITx and, more specifically, L-ITx are still not as good as those for LT and can justify proceeding to an isolated LT in particular cases [84–86]. In some very sick children, the availability of a living liver donor may also justify the decision to perform an isolated LT. Also, such an adult liver allograft might confer a greater resistance against the development of PNALD and allow more time for maximal intestinal adaptation [84–86].

The largest series published to date on isolated LT in children with IF and advanced PNALD is the experience from Omaha [84, 85]. In this series, 23 children who were estimated to have a good prognosis for nutritional independence following maximal gut adaptation received an isolated LT between 1995 and 2004. Of these 23 patients, 17 had long-term survival, with 1-year and 5-year patient survival rates of 82% and 72%, respectively, and 1-year and 5-year graft survival rates of 75% and 60%, respectively. Of those 17 patients, 14 were weaned from TPN after a median of 3 months. Of note, the majority of the patients weaned from TPN (8/14) required long-term supplemental enteral feeds to maintain adequate growth. Among the three patients who could not be completely weaned from TPN, one patient developed recurrent PNALD and required listing for L-ITx. Among the six children who died after isolated LT for PNALD, one patient died because of recurrent PNALD and four died of infectious complications. The recurrence of PNALD after isolated LT is not well defined in the literature. In one small series, three children underwent isolated LT for PNALD, none of whom were definitively weaned from TPN. Among those three patients, recurrence of PNALD after LT was seen in one patient, who ultimately died [86].

## 7. Summary

With recent improvements in immunosuppression, induction protocols, and posttransplantation management, ITx is now associated with improved patient and graft survival. The jejunoileum is the central component of every ITx. The choice of allograft is complex and every patient should undergo a multidisciplinary evaluation. ITx in patients with short gut syndrome should only be considered in those who failed dedicated intestinal rehabilitation attempts and after the development of life-threatening complications related to TPN. Inclusion of the right hemicolon should be performed in patients without full native colon in order to improve quality of life after ileostomy reversal and to optimize fluid reabsorption. In patients with PNALD, a transjugular liver biopsy should be performed and L-ITx should be considered in patients with advanced fibrosis. In patients requiring ITx for extensive portomesenteric thrombosis, this procedure should only be considered after all the other therapeutic options have failed. In the case of diffuse gastrointestinal pathology such as polyposis syndrome and motility disorders, MVTx should be considered on a case by case basis. Finally, an isolated LT can be considered in children demonstrating progressive intestinal adaptation and ESLD related to the TPN. However, when such an approach is chosen, the undefined risk of PNALD recurrence should be considered.

## Figures and Tables

**Table 1:** TPN-related complications warranting intestinal transplantation (Medicare and Medicare Services).

Impending or overt liver failure related to PNALD
≥2 episodes per year of catheter-related sepsis requiring hospitalization
≥1 episode of life-threatening infection (fungemia, septic shock, ARDS)
Impending loss of vascular access for TPN administration (thrombosis of ≥2 central veins)
Repeated episodes of significant dehydration despite IV fluids administration in supplement to TPN

*Note*. PNALD: parenteral nutrition associated liver disease; ARDS: adult respiratory distress syndrome; TPN: total parenteral nutrition.

## Abbreviations

ITx: Intestinal transplantation
IF: Intestinal failure
TPN: Total parenteral nutrition
PNALD: Parenteral nutrition associated liver disease
ITR: Intestinal transplant registry
SB: Small bowel
IITx: Isolated intestinal transplantation
L-ITx: Combined liver-intestine transplantation
MVTx: Multivisceral transplantation
SMA: Superior mesenteric artery
MMVTx: Modified multivisceral transplantation
GLP: Glucagon-like peptide
IVC: Inferior vena cava
ACR: Acute cellular rejection
SRTR: Scientific registry of transplant recipients
UNOS: United network for organ sharing
ESLD: End-stage liver disease
LT: Liver transplantation
CIPO: Chronic intestinal pseudoobstruction
GVHD: Graft versus host disease.
